# Application of Community-Engaged Research to Inform the Development and Implementation of a Peer-Delivered Mobile Health Intervention for Adults With Serious Mental Illness

**DOI:** 10.2196/12380

**Published:** 2019-03-19

**Authors:** Karen Fortuna, Paul Barr, Carly Goldstein, Robert Walker, LaPrincess Brewer, Alexandra Zagaria, Stephen Bartels

**Affiliations:** 1 Department of Psychiatry Dartmouth College Lebanon, NH United States; 2 Brown University Providence, RI United States; 3 Massachusetts Department of Mental Health Boston, MA United States; 4 Department of Cardiovascular Medicine, Mayo Clinic College of Medicine Rochester, MN United States; 5 Dartmouth-Hitchcock Lebanon, NH United States; 6 The Mongan Institute Massachusetts General Hospital Boston, MA United States

**Keywords:** stakeholder participation, mental health, patient participation, consumer advocacy, mobile health

## Abstract

**Background:**

Involving certified peer specialists in all phases of intervention development and research is a high priority to advance peer-delivered services. Certified peer specialists are individuals with a lived experience of a mental illness, and they are trained and accredited to provide Medicaid reimbursable mental health services. Community-engaged research can facilitate the development and implementation of peer-delivered interventions; however, little is known about the processes. We present our application of community-engaged research to inform the development and implementation of a peer-delivered mobile health (mHealth) intervention for adults with serious mental illness.

**Objective:**

The aim of this study was to present a framework that can be used as a guide for researchers and certified peer specialists to develop and implement peer-delivered mHealth interventions in community settings.

**Methods:**

Informed by principles of community-engaged research, we developed the Academic Researchers-Certified Peer Specialists mHealth Research Continuum. Principles of community-engaged research included in the Continuum include the following: (1) develop a clear understanding of the purpose, goal, and population involved in community change; (2) become knowledgeable about all aspects of the community; (3) interact and establish relationships with the community; (4) encourage community self-determination; (5) partner with the community; (6) respect community diversity and culture; (7) activate community assets and develop capacity; (8) maintain flexibility; and (9) commit to long-term collaboration.

**Results:**

Overall, 4 certified peer specialists participated in all phases of intervention development and research. Individuals who participated in the Academic Researchers-Certified Peer Specialists’ mHealth Research Continuum collaborated on 5 studies advancing peers’ roles in services delivery using mHealth and secured grant funding from a foundation to sustain their study. The Academic Researchers-Certified Peer Specialists’ mHealth Research Continuum has created a rare environment of inclusion by combining scientific expertise and certified peer specialists’ expertise to achieve a shared vision.

**Conclusions:**

This study delineates a process by which academic researchers and certified peer specialists participated in community-engaged research to develop and implement peer-delivered mHealth interventions in community settings.

## Introduction

### Background

Consumers diagnosed with a serious mental illness (SMI) have been long-established advocates for transparency and full partnerships with providers in treatment settings [1]. These partnerships aim to ensure human dignity, self-determination, and civil rights of consumers with SMI [1]. Despite advocacy efforts, a recent systematic review of peer-delivered intervention studies suggests that certified peer specialists do not significantly interact or assist in intervention development and implementation in a role beyond the interventionists [2]. To our knowledge, this is the first report of a community-engaged research framework that includes certified peer specialists in all stages of research and intervention development. Peers or certified peer specialists are people with a lived experience of a mental illness and have been accredited by the state to provide mental health services such as peer support [3]. Certified peer specialists are part of a national network that offers Medicaid reimbursable peer services in 34 states [4]. Consistent with the national research agenda to advance peer-delivered services [4], we present a community-engaged research framework that includes certified peer specialists in all research stages [4]. This framework can be used as a guide to participate in community-engaged research to develop and implement peer-delivered mobile health (mHealth) interventions in community settings.

Community engagement is defined as “the process of working collaboratively with and through groups of people affiliated by geographic proximity, special interest, or similar situations to address issues affecting the well-being of those people” [5]. A systematic review of community engagement found that most studies that include community-engaged research have a positive impact on health behaviors, such as diet and exercise, as well as health outcomes (eg, obesity, mental well-being, and quality of life). Principles of community engagement set forth by the Clinical and Translational Science Awards Consortium include the following: (1) develop a clear understanding of the purpose, goal, and population involved in community change; (2) become knowledgeable about all aspects of the community; (3) interact and establish relationships with the community; (4) encourage community self-determination; (5) partner with the community; (6) respect community diversity and culture; (7) activate community assets and develop capacity; (8) maintain flexibility; and (9) commit to long-term collaboration [5].

In developing peer-delivered interventions, certified peer specialists represent both interventionists’ *and* the populations’ interests. Certified peer specialists, unlike most researchers, have unique expertise and insight into the mental health care system as they have a lived experience utilizing this system for their personal health care needs [6]. Certified peer specialists have the potential to be instrumental members of research teams during all phases of developing behavioral interventions—from idea conception to effectiveness testing. For example, certified peer specialists voice ideas, concerns, and priorities that may not be part of researcher-driven intervention development and implementation. Peers can give guidance on intervention development and research procedures that are acceptable to certified peer specialists, consumers with SMI, and organizations. For example, academic researchers are commonly interested in medical outcomes; however, consumers with SMI and certified peer specialists have expressed an interest in personal recovery outcomes such as hope and empowerment. In addition, incorporating certified peer specialists as equal partners in intervention development and implementation can potentially enhance intervention success [7]. For example, social influences on health are complex and go beyond biologic and health care system factors and include social networks and support systems as well as physical environments [8]. As such, including certified peer specialists can offer valuable perspectives and insights into addressing the needs of similar populations within the context of their social and physical environments.

### Objective

Informed by community-engaged research principles, we delineate a process by which certified peer specialists were included as full partners in the development of a peer-delivered mHealth for adults with SMI. The goal of this report is to present a framework that can be used as a guide for researchers and certified peer specialists to develop and implement peer-delivered mHealth interventions in community settings.

## Methods

### Preliminary Research Before Active Engagement With the Peer Community

#### Defining the Problem From the Peers’ Perspective

Aligned with the first principle of community-engaged research, academic researchers developed a clear understanding of the social problem experienced by consumers with SMI that certified peer specialists wanted to address [5]. To begin, academic researchers conducted a community assessment using a national survey with 267 certified peer specialists from 38 states. The survey was designed to identify the top 3 biological, psychological, social, or environmental issues confronting people with SMI (Fortuna et al, unpublished data). We used an online survey to engage certified peer specialists with diverse socioeconomic backgrounds to better understand the community’s collective needs. This survey identified the management of mental health and chronic physical health conditions as a major, unaddressed issue (Fortuna et al, unpublished data).

Aligned with the principle of self-determination in community engagement, academic researchers worked with the community’s goal [5] to address mental health and chronic physical health conditions among consumers with SMI. Self-determination theory suggests that individuals have the choice and the right to determine their future [5]. Within community-engaged research, academic researchers do not have the right to impart academic research needs on the community. The concept of self-determination is consistent with the National and State Peer Support Code of Ethics [9]. Self-determination within community-engaged research is the impetus for community partners to engage in community research [5]. For example, if community members recognize and see value in addressing the problem identified and if they feel they have an influence in decision-making and can make an impact, there is a greater likelihood they will engage. In developing the partnership, academic researchers identified addressing mental health and chronic physical health conditions among people with SMI as the primary goal of the partnership. As such, over the next year, our partnership developed and tested an mHealth intervention designed to address mental and physical health self-management skill development.

#### Academic Researcher Capacity Building

Aligned with the principle of community engagement to become knowledgeable about the community [5], academic researchers developed knowledge of certified peer specialists. Academic researchers’ capacity building began by learning about peers’ beliefs, values, and culture through understanding the history of the mental health care system, reading literature written by peers (eg, *Reaching Across: Mental Health Clients Helping Each Other* [10]), and understanding the Medicaid reimbursement system for certified peer specialists. Although this process was important during early phase knowledge building, working one-on-one with certified peer specialists was the most valuable learning process.

##### Mapping Certified Peer Specialists’ Assets

Next, researchers identified certified peer specialists’ assets. For example, certification as a peer specialist in Massachusetts requires active participation in treatment; completion of an 80-hour training including classes, small group activities, and homework on fundamentals of peer support; cross-cultural partnering; use of first-person, nonclinical language; and passing score on a written examination. Certified peer specialists are then accredited to provide Medicaid reimbursable services in 34 states [4]—most commonly peer support or wellness. As such, there is potential for national dissemination and uptake if the intervention is successful.

In addition, certified peer specialists are a trained workforce with professional practice standards that could guide intervention development. Professional practice standards include (1) not forcing people to participate in services, (2) sharing stories of recovery, (3) not judging others, (4) embracing diversity, (5) educating and advocating for others, (6) addressing difficult issues, (7) learning from people they support and those supported learn from them, (8) embracing equality, (9) using a strengths-based approach, (10) setting clear expectations, and (11) focusing on the person and encouraging them to achieve what they want in life [9].

**Figure 1:**
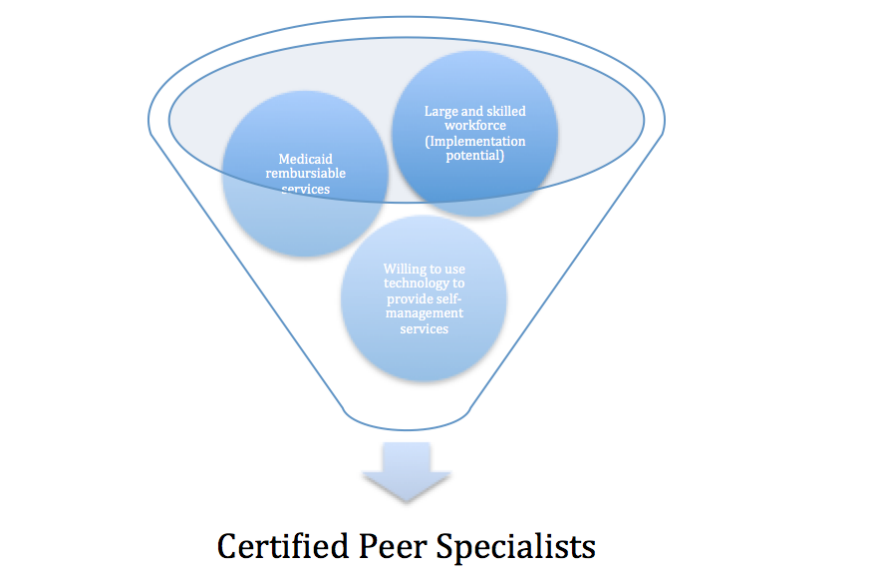
Community map of certified peer specialists’ assets.

Finally, nearly all certified peer specialists in the national online survey owned a smartphone (94.8%; 253/267), and everyone indicated that smartphones and tablets could enhance the services they deliver [11]. They reported being willing to deliver smartphone interventions for mental and physical health self-management, suggesting that smartphones may be a useful tool for offering evidence-based care (see Figure 1 for a community map of certified peer specialists’ assets).

##### Potential Power Differential

Next, academic researchers had the opportunity to informally speak with a certified peer specialist who provided services in the Massachusetts area. The purpose of this conversation was to learn more about certified peer specialists. This in-person conversation lasted approximately 30 min. As a result of this informal discussion, academic researchers advanced their knowledge of certified peer specialists beyond written material of peers’ beliefs, values, culture, and history and identified potential power differentials that may impact the relationship. For example, academic researchers learned that peers may be skeptical of people involved in the mental health care system and research—potentially due to historical, structural oppression, and stigma [12]. As such, academic researchers were led by classic and contemporary literature on social justice to assuage this potential power differential.

As academic researchers moved toward formalizing the partnership (see next section: Early Phase Research Aimed to Foster Certified Peer Specialist Engagement), principles of fairness, empowerment, inclusion, and self-determination [12-15] were introduced at the forefront of discussions between academic researchers and certified peer specialists. Specifically, academic researchers defined these principles in accordance with well-established definitions [12-15] *with* certified peer specialists. Then, in an open discussion, academic researchers and certified peer specialists elaborated on these principles and offered examples of how each principle would apply to the partnership. In Textbox 1, we present how the principles of fairness, empowerment, inclusion, and self-determination were incorporated into partnership. The inclusion of these principles formalized a set of guidelines for how the partnership would operate.

### Early Phase Research Aimed to Foster Certified Peer Specialist Engagement

#### Establishing Relationships and Developing Trust

Aligned with the principle of community-engaged research to establish an authentic relationship with the certified peer specialists’ community [5], academic researchers established a relationship and developed trust. To establish relationships with peer leaders, we developed the first iteration of the mHealth intervention with peers as consultants. The academic researchers contributed scientific knowledge to the design and development of integrated medical and psychiatric self-management interventions and expertise with research methodologies. The academic researchers identified integrated illness management and recovery (I-IMR) as having clinical effectiveness [16] and the possibility of being delivered by certified peer specialists.

**Inclusion of principles of fairness, empowerment, inclusion, and self-determination in mobile health development and implementation.**

**Fairness**

Resource sharing: financial incentives, offering transportation assistance, and food; and resource allocation: equitable pay for certified peer specialist

**Empowerment**

Peer training in the research capacity building and de-emphasized that researchers were the experts; rather, both groups brought their unique expertise to the team

**Inclusion**

Full inclusion on research teams and equal credit for mobile health intervention development as evidence by peer-reviewed publications and national presentations with peers as authors or co-presenters

**Self-determination**

Academic researchers worked with the community’s goal and modified the intervention to include peer support in addition to medical and psychiatric self-management skills training and refocused the intervention from the medical model to the recovery model of services delivery

Certified peer specialists did not assist in selecting this intervention to adapt. I-IMR is an evidence-based approach consisting of an individually tailored program addressing physical and mental health self-management in adults with SMI aged over 50 years. The academic researchers selected an intervention for older adults with SMI, as older adults with SMI are more likely to have multiple comorbidities [17] and, thus, a higher need for self-management of both medical and psychiatric conditions. Key to I-IMR is its delivery by a masters-level provider and a nurse over an 8- to 10-month period. There is evidence demonstrating that I-IMR results in improved self-management and decreased hospitalizations in older adults with SMI [16].

The academic researchers adapted I-IMR for in-person delivery by a certified peer specialist. To support fidelity, we included the use of guided eModules (ie, guided curriculum) and a smartphone app designed to complement in-person eModule sessions.

##### eModules

The eModules were designed to be reviewed on a tablet side-by-side with a certified peer specialist and a consumer with SMI during weekly 1-hour in-person sessions in a community setting. Each eModule includes videos and experiential learning tasks on psychoeducation and coping skills training. Academic researchers worked with certified peer specialists and filmed peer-led self-management videos on personal recovery stories (unscripted) that were included in the eModules. eModule sessions include (1) Identifying Your Individual Recovery and Wellness Goals: Setting recovery and health goals and strategies to achieve goals and orientation to the smartphone app; (2) Psychoeducation: Psychoeducation on SMI and medical illness; (3) Stress Vulnerability and Illness: Causes of mental illness and factors that influence its course; (4) Building Social Supports and Recovery and Wellness: How to build social supports; (5) Medication Adherence Strategies: Behavioral tailoring and motivational techniques for medication adherence; (6) Psychiatric and Medical Relapse Prevention: Identify warning signs and develop a relapse prevention plan; (7) Coping with Psychiatric Symptoms and Health-related Stress and Solving Problems: Establish a method managing symptoms; (8) Coping with Stress, Chronic Pain, and Medical Symptoms: Identifying stressors that exacerbate symptoms and strategies to cope with stress; (9) Medication Misuse: Addressing medication misuse and the effects on symptoms and functioning; and (10) A Guide to Navigating the Mental Health and Medical Health care System: Accessing mental health and medical health services and making informed decisions.

##### Smartphone App

The smartphone app was designed to assist in the transfer of self-management skills from in-person sessions with certified peer specialists to real-world environments. As we were developing the smartphone app, we had an *informal* relationship with 2 certified peer specialists and 2 consumers with SMI. We consulted with these individuals, but they were not part of the scientific team meetings. Our process included asking a clinical social worker to talk to certified peer specialists and consumers with SMI to get their opinions on the features of the smartphone app, but not the content of the intervention. The scientific team consisted of clinical social workers, physicians, and engineers [18]. The app includes personalized (1) homework from in-person meetings, (2) a relapse prevention plan, (3) daily self-management to-do checklist, (4) videos and animations to guide individuals in practicing self-management skills, and (5) articles on self-management. Branching algorithms built into the app technology allow personalization of these features to meet an individual’s personalized recovery goals. The smartphone app also included a chat feature to allow text messaging between certified peer specialists and consumers.

Once we developed these products (app and eModules), we used a scientific approach to allow peers and consumers to evaluate our study and be involved in early technology development. Academic researchers used an adaptive systems engineering approach [19] to conduct a usability test and task analysis (ie, if consumers could use the technology on their own) [18]. The usability test and task analysis was a formal scientific study, in which peers were prompted to provide continuous verbal feedback while using the app and eModules [18]. Peers were free to report whatever they felt relevant. Peers were asked to provide their verbal reactions as they completed tasks on the smartphone (app) and the tablet (eModules). Peers were asked to complete the following tasks: (1) turning the phone and tablet on, (2) finding the icon to launch the program, (3) selecting treatment programs and progressing through each program of the intervention, (4) watching videos, (5) writing and sending a text message using the text message feature within the Web app, (6) finding and checking off daily tasks, (7) reading text on the instruction page, (8) responding to a push notification prompting the participant to watch a video, and (9) returning to the homepage. Peers were asked to engage in all components of the app and eModules and provide feedback on the content, language, layout, colors, typeface, videos, graphics, text size, readability, and navigation features.

During this process, certified peer specialists provided their expertise on the needs of the community and contributed to the context of unhealthy behaviors and poor management of mental and physical health conditions. For example, academic researchers learned that healthy eating is a challenge for individuals with SMI as they may face difficulties with affording healthy foods on a limited income or due to unemployment or underemployment, or they may lack cooking skills or skills to determine healthy eating, lack a reliable place to cook, or they may eat junk food as a means to feel better in the short term. As part of this process, certified peer specialists also identified barriers and facilitators to mHealth implementation and made recommendations to ease intervention uptake. For example, certified peer specialists informed academic researchers that the app should not only be available on a smartphone but also on a tablet because older adults with SMI who wear glasses may experience difficulty reading the small text on the smartphone [18].

At this time, we learned of the need to consider certified peer specialists’ preferences and philosophy of services delivery, social and environmental contexts, and perspectives of feasibility and acceptability from certified peer specialists and consumers with SMI. We needed certified peer specialists to have a more substantive and egalitarian role in the technology development process; we needed to establish a formal partnership and to include certified peer specialists and consumers with SMI in the expert team meetings to move this program of research forward.

### Full Academic Researchers-Certified Peer Specialists Partnership

#### Establishment of a Formal Academic Researchers-Certified Peer Specialist Partnership

Aligned with community-engaged research principles, academic researchers partnered with the certified peer specialist community [5]. We engaged a community gatekeeper to develop a certified peer specialist team to guide the next phases of mHealth intervention development. Community gatekeepers are influential members of the community of interest and provide access to the community [20]. We were introduced to the *Consumer* Engagement *Liaison* for the Department of Mental Health in the state of Massachusetts. As the *Consumer* Engagement *Liaison (ie,* community gatekeeper) *understood the needs of the local community, the Liaison advocated for academic researcher inclusion in the certified peer specialists’ network within the* public mental health service system *.* This community gatekeeper model allowed ease of access and the opportunity to develop a trusting relationship between *academic researchers* and certified peer specialists.

With the assistance of the *Consumer* Engagement *Liaison, we* convened a meeting with certified peer specialists, social workers, disability rights advocates, health services researchers, primary care providers, and the Massachusetts Department of Mental Health. We discussed consumers’ needs with SMI. Together, we established a need for an effective, easily accessible self-management intervention for adults with SMI. Academic researchers presented the adapted version of I-IMR, including the eModules and the smartphone app, newly renamed as PeerTECH. After this initial meeting, a formal research partnership developed with the joint goal of assessing PeerTECH’s feasibility, acceptability, and effectiveness. Overall, 4 certified peer specialists, 2 social workers, 1 disability rights advocate, 3 health services researchers, 2 primary care providers, and 1 individual with the Massachusetts Department of Mental Health participated in all phases of intervention development and research.

#### Developing an Infrastructure for Full Participation and Shared Decision-Making Authority

Throughout this process, academic researchers respected certified peer specialists’ diversity and culture [5]. We held a series of meetings throughout the pilot study to create an infrastructure that would encourage openness and change (ie, 3 in-person and 13 virtual weekly 1-hour meetings over a 20-week period). We structured the academic researchers-certified peer specialists’ mHealth research group into 2 teams: the scientific team and the peer direct service team. The scientific team met weekly to discuss coordinating the pilot study. The scientific team included 2 people who represented organizational leadership within the selected research site, the community gatekeeper who represented certified peer specialists and consumer interests, 3 social workers familiar with consumer research participants, a peer supervisor (who was also a certified peer specialist), and 2 academic researchers (see Multimedia Appendix 1).

#### Certified Peer Specialists’ Research Capacity Building

Aligned with the principle of community-engaged research to develop community capacity, academic researchers worked with certified peer specialists to develop peers research capacity [5]. To prepare certified peer specialists to be involved in research, the principal investigator met with certified peer specialists on the scientific team and also the peer direct service team. Orientation included an open discussion of the current state of the evidence; models for developing behavioral interventions; intervention components; the role of peer interventionists and the peer researcher; and defining expectations of a culture of openness, trust, respect, commitment, flexibility, adaptation, and willingness to compromise. Capacity building included training on the following: (1) research terminology such as pre-posttests, pilot, and outcome measurement; (2) research procedures such as participant safety, informed consent, and data collection; (3) working collaboratively; (4) shared decision-making; and (5) respecting diversity. Instructional methods included experiential learning, role-play, and teach-back method. Academic researchers solicited feedback on how to improve research training.

Once trained, certified peer specialists directed scientific efforts related to the following: (1) text message dose; (2) recruitment—deciding on the location, identifying and hiring, training certified peer specialists using academic profiling, and implementation; (3) identifying outcomes of interest; (4) modified research questions (see below for detailed description); (5) resource allocation—defining equitable pay for certified peer specialists, caseload, and hours required; (6) interpretation of the findings; and (7) dissemination.

The peer direct service team met weekly with the peer supervisor (ie, the peer supervisor was also included in the scientific team) to discuss services delivery, training needs, and modifications to PeerTECH intervention delivery and management procedures. The peer supervisor directed issues to academic researchers weekly during the collaborative research team meetings. Issues brought forth by the peer supervisor included transportation for certified peer specialists, developing a program for matching peers with consumers, and technology training for certified peer specialists. Pragmatic considerations in community-engaged research included flexibility and resource sharing, for example, during training, we included providing financial incentives to certified peer specialists, flexibility with starting times and overestimating timeline, and offering transportation assistance and food.

### Ongoing Opportunities for Academic Researchers-Certified Peer Specialists’ Co-Learning

#### Reciprocal Capacity Building and Learning

We included reciprocal capacity building and co-learning in our application of community-engaged research. On the basis of certified peer specialists’ expertise, peers advocated to examine additional outcomes and also identified potential mechanisms of action. Specifically, peers identified important outcomes including social support, hope, and empowerment. As such, we examined these outcomes. Peers also suggested that without having hope, how is managing ones’ mental and physical health possible? Thus, we defined hope as a mechanism of action on the self-management of medical and psychiatric skill development. As such, academic researchers and certified peer specialists also modified the existing research questions. The original research question included the following: “to what extent does PeerTECH impact self-management skill development?” This research question was modified to examine “to what extent does PeerTECH impact self-management skill development, hope, empowerment, and social support?”

Certified peer specialists worked alongside academic researchers and identified research sites and acceptable screening tools and assisted in hiring, training, and managing peers. The academic researchers-certified peer specialists’ mHealth research group allowed reciprocal capacity building and learning. For example, researchers learned peer history and services delivery practice standards, peer support, and mutuality. The major change this made to the research included modifying the intervention to include peer support in addition to medical and psychiatric self-management skills training, refocusing the intervention from the medical model to the recovery model of services delivery, and the inclusion of consumer-reported measures. In practice of cultural humility (ie, a process of self-reflection that supports individuals in learning about others’ and their own beliefs and identity [20]), we de-emphasized that researchers were the experts; rather, both groups brought their unique expertise to the team. Cultural humility allowed academic researchers to accept and maintain personal flexibility to allow rapid intervention co-design—consistent with the principles of community-engaged research.

## Results

### Rapid Iterative Intervention Co-Design

The academic researchers-certified peer specialists’ mHealth research group led the effort to examine the feasibility, acceptability, and preliminary effectiveness of PeerTECH with adults with SMI in a pre-post pilot study. Our study design and findings have been reported elsewhere [21]. PeerTECH showed statistically significant improvements in psychiatric self-management on the illness management and recovery scale (IMRS) [22]. The IMRS is a valid, reliable 15-item scale that assesses domains of illness management [22]. Each item addresses psychiatric illness, management, and recovery. Although we were not powered to detect statistically significant differences, improvements were found including positive changes in self-efficacy for managing health conditions, hope, quality of life, medical self-management skills, and empowerment.

At the pilot study’s conclusion, we conducted a focus group with 3 certified peer specialists involved with PeerTECH and 8 individual interviews with adults with SMI [23]. Both groups agreed technology was a vital component of PeerTECH that allowed health behavior change, self-management therapeutic techniques, engagement in health technology, and peer support [23]. Peers assisted in redesigning the curriculum and requested PeerTECH to promote certified peer specialists’ professional practice standards, including (1) sharing peers’ recovery story, (2) reciprocal learning between peers and consumers whom they support, (3) focusing on the person and their personal goals, and (4) include additional modules on hoarding and trauma-informed care. Certified peer specialists and academic researchers also identified implementation barriers and facilitators to using mHealth in environmental contexts. Barriers identified included text size and the need for the app to be available on a tablet. In response, we are redesigning PeerTECH with the academic researchers-certified peer specialists’ mHealth research group and planning a randomized control trial. In addition, technology allowed everyone to be engaged, regardless of timing and physical barriers.

#### Engaging Certified Peer Specialists to Cofacilitate Dissemination

Aligned with the community-engaged research principle of activating community assets [5], academic researchers engaged certified peer specialists to cofacilitate dissemination. Certified peer specialists and other stakeholders have been instrumental in disseminating study results through national presentations and peer-reviewed publications. Our research team has historically published and presented with certified peer specialists at national and state conferences. In addition to academic researchers’ dissemination efforts, we utilize other opportunities of dissemination including social media such as Facebook, LinkedIn, and Twitter. To ensure the peers and consumers are informed of our findings, we have worked with certified peer specialists to help us translate the study findings for social media posts to reach a broader audience.

#### Long-Term Collaboration Between Academic Researchers and Certified Peer Specialists

Aligned with the principle of community-engaged research to commit to long-term collaboration [5], academic researchers and certified peer specialists created a sustainable workgroup. Before the development of the academic researchers-certified peer specialists mHealth research group, the Dartmouth Centers for Health and Aging had no formal research collaborations with certified peer specialists. Although the Dartmouth Centers for Health and Aging has a history of delivering consumer co-led services, certified peer specialists were not involved in the development of these interventions nor were they on research teams. It was essential to work with certified peer specialists and create an infrastructure that would facilitate openness, trust, and respect between academic researchers and certified peer specialists.

Individuals in the academic researchers-certified peer specialist mHealth research group have collaborated on 5 studies over 1 year to advance the role of peers in services delivery using mHealth. We anticipate this group to be long-term as it has secured grant funding from a private foundation to sustain their study. Consistent with principles of community engagement, this approach to community-engaged research has created a rare environment of inclusion through the combination of scientific expertise and certified peer specialists’ expertise (see the Academic Researchers-Certified Peer Specialists mHealth Research Continuum in Multimedia Appendix 1).

## Discussion

### Principal Findings

This is the first report of a community-engaged research framework that includes certified peer specialists in all stages of research and mHealth intervention development. Unlike the recent models of mHealth community-engaged research that focus on only *1* aspect of community engagement (ie, usability testing), we incorporated peers in all aspects of research and mHealth intervention development. In our framework, peers were equal partners in helping define the problem, creating mHealth intervention content, identifying outcomes of interest, modifying research questions, and identifying research sites, and they also assisted researchers in hiring, training, and managing peers. Finally, peers also guided our dissemination efforts. Our framework can be used as a guide for researchers and certified peer specialists to develop and implement peer-delivered mHealth interventions in community settings.

### Conclusions

mHealth intervention development and implementation is a complex scientific process that incorporates multiple disciplines with their own distinctive cultures and expertise [18]. Promoting empowerment and autonomy with certified peer specialists can be a challenging process in addition to an already complex undertaking. For example, including consumers in mHealth intervention development requires increased time and resources to facilitate equal partnerships, including developing trusting relationships and capacity building; understanding consumers’ opinions, culture, and philosophies; addressing potential relapse; and building and maintaining equal, respectful partnerships. Despite this complex undertaking, we posit that peer-delivered mHealth interventions designed to improve the lives of people with SMI include certified peer specialists with lived experience as experts in every mHealth intervention development and implementation phase.

## Appendix

Multimedia Appendix 1 Framework for the Academic Researchers-Certified Peer Specialists mHealth Research Continuum designed to simultaneously inform the development and implementation of a peer-delivered mHealth intervention for adults with serious mental illness.

## Abbreviations

I-IMR: integrated illness management and recovery
IMRS: illness management and recovery scale
mHealth: mobile health
NIH: National Institutes of Health
SMI: serious mental illness
