# Supplementary material for: Application of Community-Engaged Research to Inform the Development and Implementation of a Peer-Delivered Mobile Health Intervention for Adults With Serious Mental Illness
Source: J Particip Med. 2019 Mar 19;11(1):e12380. doi: 10.2196/12380 (PMC7039401; doi:10.2196/12380)
Supplement: Multimedia Appendix 1 [file jopm_v11i1e12380_app1.pdf]

**Preliminary Research before Active Engagement with Peer Community**	**Early Phase Research Aimed to Foster Peer Engagement**	**Full Academic Researchers-Certified Peer Specialists Partnership**	**On-going Opportunities for Academic Researchers and Certified Peer Specialists' co-learning**	**Sustainability**
**KNOWLEDGE BUILDING**	**CONSULTATION**	**FULL PARTNERSHIP**	**CO-LEARNING**	**LONG-TERM COLLABORATION**
**Defining the problem from peers' perspective**<br>*National online survey*<br><br>**Principles of community engagement:**<br>(1) Develop a clear understanding of the purpose, goal and population involved in community change; (2) Community self-determination	**Establishing relationships and developing trust**<br>*Academic researchers developed the first iteration of the mHealth intervention with certified peer specialists as consultants.*<br><br>**Principles of community engagement:**<br>Interact and establish relationships with the community	**Establishment of a formal academic-peer partnership** *A community gatekeeper advocated for academic researchers' inclusion in the certified peer network—allowing for ease of access and the opportunity to develop a trusting relationship.*<br><br>**Principle of community engagement:**<br>Partner with the community	**Reciprocal capacity building and learning**<br>*Certified peer specialists assist in the modification of research questions, adding assessment measures, and identifying mechanisms. Certified peer specialists guided effort identifying research sites, acceptable screening tools, hiring, training, and managing peers.*<br><br>**Principle of community engagement:**<br>We expanded our application of community-engaged research to include reciprocal capacity building and co-learning	**Long-term Collaboration**<br>*Creation of a rare environment of inclusion through the combination of scientific expertise and peer expertise. Grant funding for future collaborative projects was secured.*<br><br>**Principles of community engagement:**<br>Commit to long-term collaboration

**Researcher capacity building** *Map community assets and examining potential power differential.*<br><br>**Principle of community engagement:** Become knowledgeable about all aspects of the community		**Developing an infrastructure for full participation and shared decision-making authority**<br>*Formal meetings with scientific team and the certified peer specialists direct service team.*<br><br>**Principle of community engagement:**<br>Respected the community's diversity and culture	**Iterative mHealth intervention co-design**<br>*Peers assisted in redesigning the curriculum and identified implementation barriers and facilitators to using mHealth in environmental contexts.*<br><br>**Principle of community engagement:**<br>We expanded our application of community-engaged research to include reciprocal capacity building and co-learning	
		**Certified peer specialists research capacity building**<br>*Training on: research terminology and procedures; working collaboratively; shared decision-making; respecting diversity. Instructional methods included experiential learning, role-play, and teach-back method.*<br><br>**Principle of community engagement:**<br>Become knowledgeable about the community	**Engaging peers to co-facilitate dissemination**<br>*Certified peer specialists inclusion in national presentations, peer-reviewed publications, and delivering social media messages.*<br><br>**Principle of community engagement:**<br>Activate community assets	

**Action Step:**<br>1) Academic researchers conducted a national online survey of peers. Peers identified addressing mental health and chronic physical health conditions among people with SMI as a major unmet need.<br><br>2) Academic researchers defined the primary goal of the partnership as "addressing mental health and chronic physical health conditions among people with SMI"	**Action Step:**<br>1) Academic researchers selected Integrated Illness Management and Recovery as having real-world effectiveness and potential to be adapted to be delivered by peers with the assisted use of mHealth.<br><br>2) Academic researchers produce PeerTECH technologies (eModules and app) in consultation with peers.<br><br>3) Peers were featured in unscripted videos that shared their recovery story and self-management tips; videos were embedded in the app and eModules.<br><br>4) Academic researchers conducted usability tests with peers as research participants. Peers provided recommendations on changing the content and identified potential implementation barriers.	**Action Step:**<br>1) Formal meetings with academic researchers and the certified peer specialists direct service team.<br><br>2) Academic researchers developed the research training for peers.<br><br>3) Academic researchers conducted research training with peers.<br><br>4) Academic researchers solicited feedback on how to improve research training.	**Action Step:**<br>1) Conduct study with peers as equal partners:<br>-Peers assisted in the modification of research questions, adding outcomes of interest, determining intervention dose, identifying mechanisms of action, recruitment (i.e., guided effort identifying research sites, hiring, training, and managing peers), resource allocation (i.e., defining equitable pay for certified peer specialist, caseload and hours required); interpretation of the findings; and dissemination (i.e., national presentations and published peer-reviewed publications and published social media posts).<br><br>2) After conducting the study, formal meetings with the academic researcher team and the certified peer specialists	**Action Steps:**<br>1) Academic researchers found grant opportunities.<br><br>2) Academic researchers and peers applied for grants (i.e., scientists wrote grants in collaboration with peers; peers also helped secure research sites and letters of support).<br><br>3) Academic researchers and peers secured grant funding.

			direct service team led to modified mHealth intervention content and implementation procedures.	
